# Characterising the spatial and oscillatory unfolding of Theory of Mind in adults using fMRI and MEG

**DOI:** 10.3389/fnhum.2022.921347

**Published:** 2022-09-20

**Authors:** Sarah I. Mossad, Marlee M. Vandewouw, Kathrina de Villa, Elizabeth W. Pang, Margot J. Taylor

**Affiliations:** ^1^Department of Psychology, The Hospital for Sick Children, Toronto, ON, Canada; ^2^Department of Diagnostic Imaging, Hospital for Sick Children, Toronto, ON, Canada; ^3^Program in Neurosciences and Mental Health, Hospital for Sick Children, Toronto, ON, Canada; ^4^Autism Research Center, Bloorview Research Institute, Holland Bloorview Kids Rehabilitation Hospital, Toronto, ON, Canada; ^5^Institute of Biomedical Engineering, University of Toronto, Toronto, ON, Canada; ^6^Division of Neurology, Hospital for Sick Children, Toronto, ON, Canada; ^7^Departments of Psychology and of Medical Imaging, University of Toronto, Toronto, ON, Canada

**Keywords:** Theory of Mind (ToM), functional magnetic resonance imaging (fMRI), magnetoencephalography (MEG), social attribution, functional connectivity

## Abstract

Theory of Mind (ToM) is a core social cognitive skill that refers to the ability to attribute mental states to others. ToM involves understanding that others have beliefs, thoughts and desires that may be different from one's own and from reality. ToM is crucial to predict behaviour and navigate social interactions. This study employed the complementary methodological advantages of both functional MRI (fMRI) and magnetoencephalography (MEG) to examine the neural underpinnings of ToM in adults. Twenty healthy adults were first recruited to rate and describe 28 videos (15s long), each containing three moving shapes designed to depict either social interactions or random motion (control condition). The first sample of adults produced consistent narratives for 6 of those social videos and of those, 4 social videos and 4 control videos were chosen to include in the neuroimaging study. Another sample of twenty-five adults were then recruited to complete the neuroimaging in MEG and fMRI. In fMRI, we found increased activation in frontal-parietal regions in the social compared to the control condition corroborating previous fMRI findings. In MEG, we found recruitment of ToM networks in the social condition in theta, beta and gamma bands. The right supramarginal and angular gyri (right temporal parietal junction), right inferior parietal lobe and right temporal pole were recruited in the first 5s of the videos. Frontal regions such as the superior frontal gyrus were recruited in the second time window (5–10s). Brain regions such as the bilateral amygdalae were also recruited (5–10s), indicating that various social processes were integrated in understanding the social videos. Our study is one of the first to combine multi-modal neuroimaging to examine the neural networks underlying social cognitive processes, combining the strengths of the spatial resolution of fMRI and temporal resolution of MEG. Understanding this information from both modalities helped delineate the mechanism by which ToM processing unfolds over time in healthy adults. This allows us to determine a benchmark against which clinical populations can be compared.

## Introduction

Humans have an advanced capacity to ascribe intentions to the minds of others. Premack and Woodruff (1978) coined the term “Theory of Mind” (ToM) to capture this ability to make inferences about the mental states of others including perspectives, desires and beliefs. During social interactions, ToM enables an individual to predict another person's behaviour. Explicit ToM is acquired between 3 and 5 years of age (Wellman et al., 2001, 2011; Callaghan et al., 2005), but ToM skills continue to improve throughout development (Blakemore, 2012; Lagattuta et al., 2015) and mastery of the subcomponents of ToM varies individually and cross-culturally (Shahaeian et al., 2011; Wang et al., 2016).

A debate remains ongoing regarding the mechanisms of ToM processing in part because it is hypothesised that ToM is not a singular skill and relies on multiple subprocesses including but not limited to self/other distinction, emotion processing, face recognition, cognitive flexibility, inhibition, working memory, moral reasoning, etc. as proposed by Schaafsma et al. (2015). Secondly, the term ToM remains unclearly operationalized across studies, with a range of paradigms that often focus on a single specific subtype of ToM such as inferring a character's perceptions, emotions or cognitive states, making it difficult to compare directly across studies. Despite these methodological constraints, fMRI investigations have furthered our understanding of a network of brain regions that is consistently activated across various ToM tasks (Carrington and Bailey, 2009; Schurz et al., 2014; Kliemann and Adolphs, 2018; Arioli et al., 2021). The right temporal-parietal junction (an area encompassing the angular and supramarginal gyri) is thought to be central to ToM processing due to its selective activation in mental state attribution conditions compared to social descriptions (Saxe and Wexler, 2005). Others have argued that the medial prefrontal cortex is most crucial, by serving a central role in social cognition, and specifically in thinking about oneself and others (Gallagher et al., 2000; Amodio and Frith, 2006). The right TPJ is associated with re-orienting to others and inferring their mental states (Saxe and Kanwisher, 2003; Rothmayr et al., 2011), and the medial prefrontal cortex is involved in decoupling thoughts about self from thoughts about others (Döhnel et al., 2012; Schuwerk et al., 2014).

Although the fMRI literature has advanced our knowledge of the key players in the ToM network, our understanding of how these regions *functionally* communicate remains unclear in the ToM neuroimaging literature. Other neuroimaging modalities such as EEG have provided some information about the timing of activation of these brain areas. For example, an early event-related potential (ERP) study using a subtype of ToM tasks known as false belief, showed increased late positive complex (LPC) over parietal regions (300–600ms) followed by a late slow wave divergence over anterior regions (600–900ms) in adults in the false belief compared to the true belief condition (Meinhardt et al., 2011). MEG (magnetoencephalography) can measure neuronal activation with far superior temporal resolution to fMRI, with access to information about the *timing* of activation (in ms), as well as *oscillatory* activity as it can directly quantify the brain's neural activity (Hari and Salmelin, 2012) and is not distorted by the skull or scalp, providing better spatial resolution than EEG. MEG allows the quantification of local and long-range oscillatory changes and their localisation in the brain (Hunt et al., 2019).

Previous MEG connectivity studies have shown that cognitive processes are modulated by specific frequency oscillations including theta (implicated in long-range communication in the brain), alpha (implicated in several cognitive processes including attention and memory), beta (implicated in top down processing) and gamma (implicated in visual attention, awareness, emotional processing) (Von Stein and Sarnthein, 2000; Engel and Fries, 2010; Palva et al., 2010; Klimesch, 2012; Mellem et al., 2013; Sherman et al., 2016; Solomon et al., 2017; Betti et al., 2018; Richter et al., 2018; Soto-Icaza et al., 2019). Although the current fMRI literature suggests that ToM is supported by a core ToM network, whether there are multiple independent networks which are temporally separate and modulated by different frequency bands remains a question. This is particularly important since ToM difficulties are common in several clinical populations such as autism spectrum disorder, schizophrenia, preterm birth and traumatic brain injury (Baron-Cohen et al., 1985; Penn et al., 2002; Hill and Frith, 2003; Brüne, 2005; Chertkoff Walz et al., 2010; Martín-Rodríguez and León-Carrión, 2010; Williamson and Jakobson, 2014; Bora and Berk, 2016; Mossad et al., 2017; d'Arma et al., 2021; Csulak et al., 2022) and access to information about ToM processing in typical development allows us to investigate the various aetiologies of these ToM deficits in clinical populations. A recent study on individuals with ASD highlighted that a multimodal neuroimaging approach would likely be more helpful to study ToM in ASD as their fMRI results did not point to clear differences in the ToM network that would relate to experienced social difficulties (Moessnang et al., 2020). An MEG functional connectivity analysis would therefore allow us to further characterise (1) which regions in the core ToM network are functionally connected, (2) the temporal sequence of these network dynamics, and (3) whether specific frequency bands support different ToM networks. Thus, using these two neuroimaging modalities (fMRI and MEG) allows us to pinpoint the stages of ToM processing. We do this by relying on fMRI which has excellent spatial localisation to assess whether our findings corroborate findings from previous source localisation studies, and by relying on MEG which has excellent temporal and oscillatory resolution to determine when these regions are functionally connected, as well as whether there are particular oscillatory characteristics that underlie these brain networks.

In this study, we measured ToM ability using a social attribution task, first introduced by Heider and Simmel (1944) and further developed by others (Castelli, 2002; Schultz et al., 2003; Gobbini et al., 2007). The social attribution task involves moving shapes designed to depict social interactions and invokes mental state attributions from the viewer. This task has been used in fMRI to interrogate ToM processing (Kana et al., 2015; Martin et al., 2016; Synn et al., 2017) as it has lower cognitive demands compared to classic false belief tasks. Using the social attribution task in this study therefore allows replication in other clinical populations using multi-modal neuroimaging. Although ceiling effects can occur in the behaviourally administered version of this task, we were interested mainly in the neural correlates of the ToM condition of the task.

We recruited two groups of healthy adults to complete this study. The first group (*n* = 20) watched a set of videos depicting either social interactions or random motion and provided their verbal descriptions of each scenario. Videos where all subjects attributed mental states to the shapes were categorised as the ToM condition and videos were all the subjects did not attribute mental states to the shapes were categorised as the non-ToM condition. The second group of healthy adults (*n* = 25) completed the social attribution task in the fMRI and MEG. The study had three main objectives: (1) to corroborate previous findings that the social attribution task recruits core ToM regions in fMRI, (2) to determine which frequency bands modulate ToM processing in the social attribution task, and (3) to establish which regions of the ToM network are functionally connected. We predicted greater recruitment of the ToM network in the social compared to the control condition and further that ToM processing would be coordinated by oscillatory activity in beta band based on previous EEG findings (Guan et al., 2018).

## Methods

### Participants

Forty-five adults were recruited by posting flyers in the Peter Gilgan Centre for Research and Learning at SickKids, Toronto, Canada. Twenty of those adults were recruited to provide descriptions for 28 videos designed by Klin (2000) and Schultz et al. (2003) that depict social interactions (16 videos) or random motion (12 videos). There was consensus among participant narratives for 6/16 social videos and 6/12 random motion videos. Of those, four videos depicting social interactions and four videos depicting random motion were included in the social attribution task.

A second sample of twenty-five adults (12 females, mean age: 26.7 ± 5.4 years) completed the social attribution task in MEG and fMRI. Exclusion criteria included intellectual impairment or any other language or vision issues preventing successful completion of tasks, as well as standard MEG/MRI contraindications. This study was approved by the SickKids research ethics board. Participants gave their written informed consent according to the declaration of Helsinki. All study components, intellectual testing, MEG and fMRI scans, were completed on the same day.

### Intellectual testing

Participants completed two subtests (Vocabulary and Matrix Reasoning) of the Wechsler Abbreviated Scale of Intelligence (WASI, Wechsler, 1999) as an estimate of their Full-Scale IQ.

### The social attribution task

The social attribution task is based on previous findings that adults attribute mental states (intentions, emotions, beliefs and personality traits) to moving geometric shapes based on their kinetic features (Heider and Simmel, 1944). These findings have been replicated in various behavioural studies and later in neuroimaging studies (Phelps and LeDoux, 2005; Amodio and Frith, 2006; Hynes et al., 2011).

The task consisted of eight 15-s videos which included three white shapes (square: 0.6 × 0.6 cm, circle: 1 cm diameter, and triangle: 1.4 × 1 × 1 cm) moving in and around a white fixed square frame (with one side that hinged open and shut). The frame was centred in the middle of the screen and the shapes were displayed against a black background. The task included two conditions: a social condition and a control condition. In the social condition, the videos depicted a non-verbal narrative—with a start, middle and resolution—which unfolded among the shapes. In the control condition, the three shapes were moving randomly (at varying speeds and angles) across the screen. The basic visual characteristics (speed, orientation of motion, etc.) were similar across the social and control conditions.

**Figure 1:**
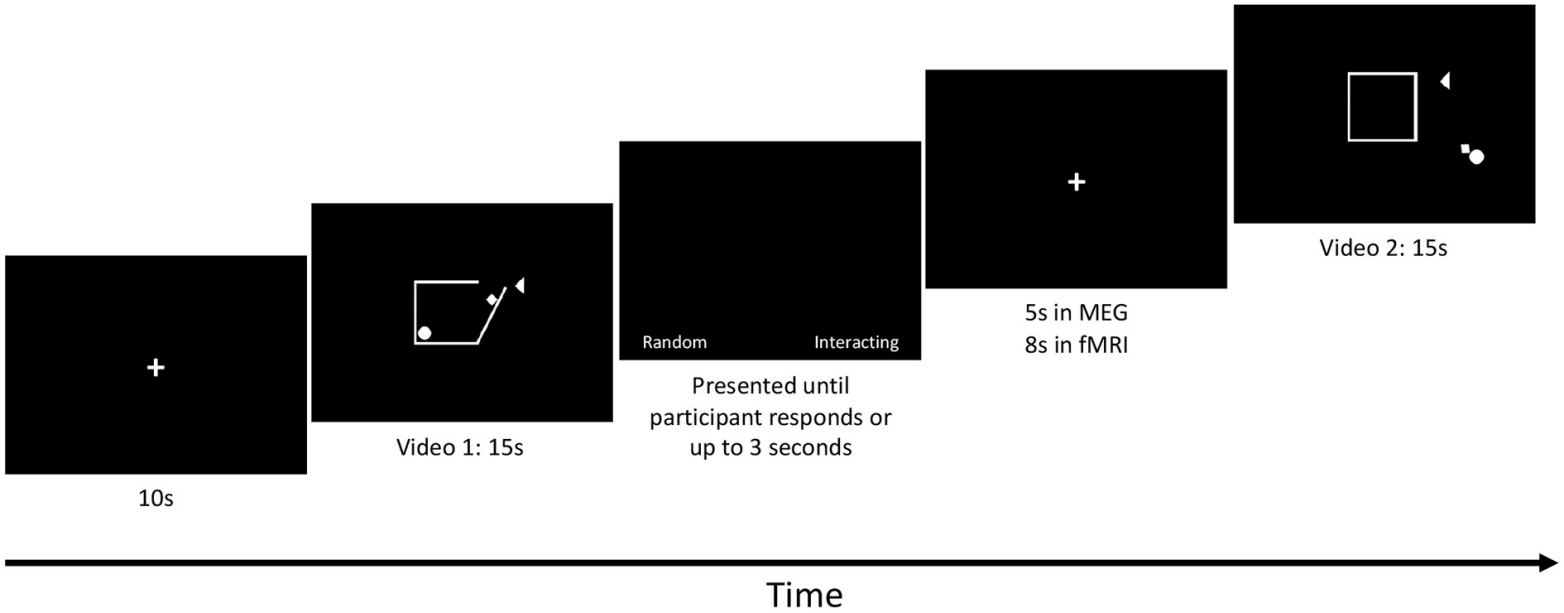
SAT protocol: Each run started with a 10-s baseline followed by a 15-s video. Each video was followed by a prompt for each subject to respond by button press to whether the shapes' movement was interpreted as Random (Control condition) or whether the movements were interpreted as Interacting (Social condition). The prompt was presented until each subject responded or up to 3 s elapsed. An inter-stimulus interval was then presented. The length of the inter-stimulus interval was 5 s in MEG and 8 s in fMRI. The videos were designed by Klin (2000) and Schultz et al. (2003).

A 10 second baseline (black screen with a white centred fixation cross) was presented at the beginning of the task in both the MEG and fMRI scans, followed immediately by the first video, Figure 1. The eight videos were presented in pseudorandom order and each video was followed by a prompt for participants to choose whether the shapes were moving randomly (“Random”) or were socially interacting (“Interacting”). This prompt included a fixation cross centred on the screen and the two words were displayed on either side of the fixation cross. Participants had 3 s to respond. Following this prompt, an interstimulus interval with only the fixation cross was presented (5 s in the MEG and 8 second in the fMRI). The task was run 3 times in both MEG and fMRI. The run time of the task was 3.3 min in the MEG and 3.7 min in the fMRI for each run.

Following the MEG and MRI scans, participants watched each video and provided verbal responses to the question “Tell me everything that is happening in this video”. Their responses were recorded on an audio recorder, transcribed and then scored based on scoring criteria developed by Klin (2000). Participants' attributions were categorised into behaviour (e.g., triangle *chases* square), perceptions (e.g., square *sees* the circle), emotions (e.g., square is *happy*), cognitions (e.g., triangle *wants* to trick the circle), relationship/personality traits (e.g., square is circle's *friend* or square is a *bully*). Participants' use of symbolic descriptions (e.g., the square and circle leave their *house*) was also scored and the mean number of attributions in between conditions was compared using paired *t-*tests.

### Data acquisition and pre-processing

#### fMRI

T1-weighed structural magnetic resonance images (MRIs) were collected on a 3T Siemens MAGNETOM Prisma Fit scanner with a 20-channel head and neck coil. A three-dimensional (3D) magnetisation-prepared rapid gradient-echo (MPRAGE) sequence (TR/TE/TI: 1870/3.14/945 ms; FA: 9°; FOV: 240 × 256 mm; number of slices: 192; resolution: 0.8 mm isotropic; scan time: 5:01 min) was used to collect the MR images.

fMRI data were acquired while participants completed the social attribution task using an echo planar imaging (EPI) sequence (TR/TE: 1,500/30 ms; FA: 70°; FOV: 222 × 222 mm; number of slices: 50; resolution: 3 mm isotropic). Participants responded using button press (on a 4-button Diamond Fibre Optic Response Pad by Current Designs).

All scanning took place at the Hospital for Sick Children (Toronto, Canada). Standard Analysis of Functional NeuroImages (AFNI, Cox, 1996; Cox and Hyde, 1997), FreeSurfer (Dale et al., 1999) and FMRIB Software Library (FSL, Jenkinson et al., 2012) tools were used to process fMRI data. T1-weighted images were skull stripped using FreeSurfer. Slice timing and motion correction were applied to the fMRI data and the 6 motion parameters (3 translations + 3 rotations) were estimated. We calculated frame-wise displacement (FD), and volumes with FD > 0.9 mm (Siegel et al., 2014) were removed from the data. We used a 6 mm FWHM Gaussian kernel to smooth data and then data were intensity normalised. White matter, CSF and whole-brain signal contributions along with the 6 motion parameters were regressed from the data. A 0.01–0.2Hz bandpass philtre was applied. FSL's FLIRT (Jenkinson and Smith, 2001) was used to register functional images to MNI standard space and FIX was used for ICA denoising (Griffanti et al., 2014; Salimi-Khorshidi et al., 2014).

#### MEG

Participants were scanned in a magnetically shielded room in supine position using a 151-channel CTF system (CTF MEG International Service LP, Coquitlam, BC, Canada). Data were recorded at a 600 Hz-sampling rate with third order noise cancellation and continuous head localisation throughout the recording. Stimuli were presented ~80 cm from the participant. Participants responded on a VPIXX 4 button pad (Visual Science Solutions, Saint-Bruno, Canada). Analyses and statistics were conducted in MATLAB implementing functions from the FieldTrip toolbox (Oostenveld et al., 2011), Network-Based Statistics (Zalesky et al., 2010), BrainNetViewer (Xia et al., 2013) and Marc's MEG Mart (MMM; https://gitlab.com/moo.marc/MMM).

“Social” and “Control” trials were epoched from −5 to 17s. Heartbeat and ocular artefacts were removed using ICA by author SIM. Trials where the signal exceeded 2,500 fT were also rejected. Head motion was calculated by fitting a rigid sphere to the average fiducial marker locations (right and left pre-auricular points and nasion) and tracking the motion (rotation and translation) of the sphere continuously using the HeadMotionTool from the M MM toolbox. Trials with >10 mm motion from the median head position were rejected.

##### MEG source analysis

Data were imported to MATLAB, mean-centred and then filtered with a 4th order Butterworth band-pass philtre from 1 to 150 Hz, as well as a discreet Fourier transform notch philtre at 60 and 120 Hz to remove line noise. Single shell head models based on each participant's MRI were computed using SPM12 through FieldTrip and template coordinates were non-linearly transformed into subject-specific coordinates. Linearly constrained minimum variance (LCMV) beamforming with 5% regularisation and projection of the activity to the dominant orientation was performed to estimate the neural activity index (NAI) at the centroid of each of the cortical and subcortical regions of the AAL atlas.

##### MEG connectivity analysis

The NAI timeseries were then filtered into 4 canonical frequency bands: theta (4–7 Hz), alpha (8–12 Hz), beta (13–29 Hz), gamma (30–55 Hz), using FIR philtres (MATLAB's fir1). Filtered NAI time series were orthogonalized (using the symmetric orthogonalization procedure from Colclough et al., 2015) to remove effects of signal leakage.

Connectivity was estimated using amplitude envelope correlations (AEC). Amplitude envelopes were computed using the absolute value of the Hilbert transform (Brookes et al., 2011; Hipp et al., 2012). To obtain the AEC, the Pearson correlation coefficient was computed for amplitude envelopes from each pair of nodes. The AEC time series were then baseline corrected by calculating the fractional change from the mean baseline AEC (−5 to 0 s).

### Statistical analysis

#### fMRI

First-level analyses using the task conditions (social, control, baseline and response) were used as explanatory variables and convolved with a double-gamma hemodynamic response function using FMRIB's Improved Linear Model (FILM; Woolrich et al., 2001). The model included nuisance regressors for the 6 motion parameters and motion-scrubbed volumes and investigated contrasts between the social and control conditions. Second-level analysis was performed to average contrast estimates over runs within each subject using FSL's FMRI Expert Analysis Tool (FEAT) with fixed effects (Woolrich et al., 2004). Finally, the across-condition effects of the social vs. control contrast were examined using FMRIB's Local Analysis of Mixed Effects (FLAME; Woolrich et al., 2004). Multiple comparisons correction was performed with Gaussian random field theory at the cluster level (*Z* > 2.3), holding significance at *p*_corr_ < 0.05. The cluster size threshold as calculated by FEAT was at least 225 voxels.

#### MEG

Whole brain network connectivity in the social condition (Social > Baseline) was identified in Network Based Statistics (NBS) (Zalesky et al., 2010). First, NBS applies mass univariate testing to test the null hypothesis at each connexion between two nodes across the whole brain. For each connexion, a strict *t-*value threshold of 2.75 was applied, allowing only connexions with a *t-*value of 2.75 and above to be included. Next, NBS examines the topology among the connexions which passed suprathreshold connexions using cluster-based statistics. Therefore, each surviving cluster was composed of supra-threshold connexions, with a path connecting any two nodes. Finally, permutation testing (permutations were repeated 5,000 times) was used to compute a family wise error corrected *p*-value for each network. Networks passing the significant threshold (*p*_corr_ < 0.05) are reported for the time windows of interest (0–5, 5–10, 10–15 s).

## Results

### Intellectual testing

Participants who completed the neuroimaging component had an average IQ of 117.1 ± 10.5.

### SAT descriptions

**Figure 2:**
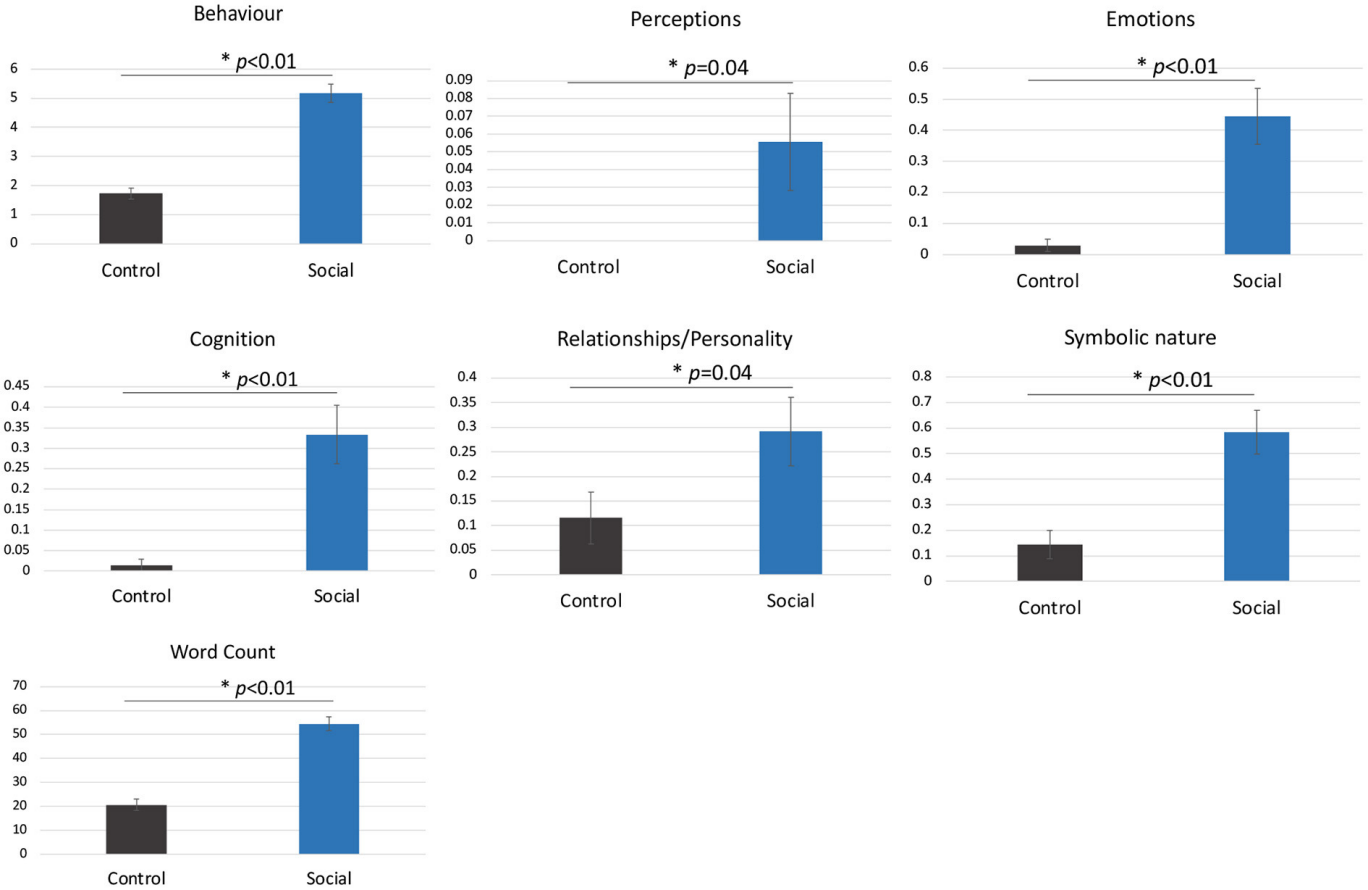
The mean number of attributions per video in the Social compared to Control condition (± standard error). Asterisks represent a statistically significant difference. Participants did not make perceptual attributions in the Control condition.

Participants made significantly more attributions overall to the shapes in the Social compared to Control condition including more behavioural, emotional and cognitive state attributions (all *p*s < 0.05, Figure 2).

### Neuroimaging results

#### fMRI results

**Figure 3:**
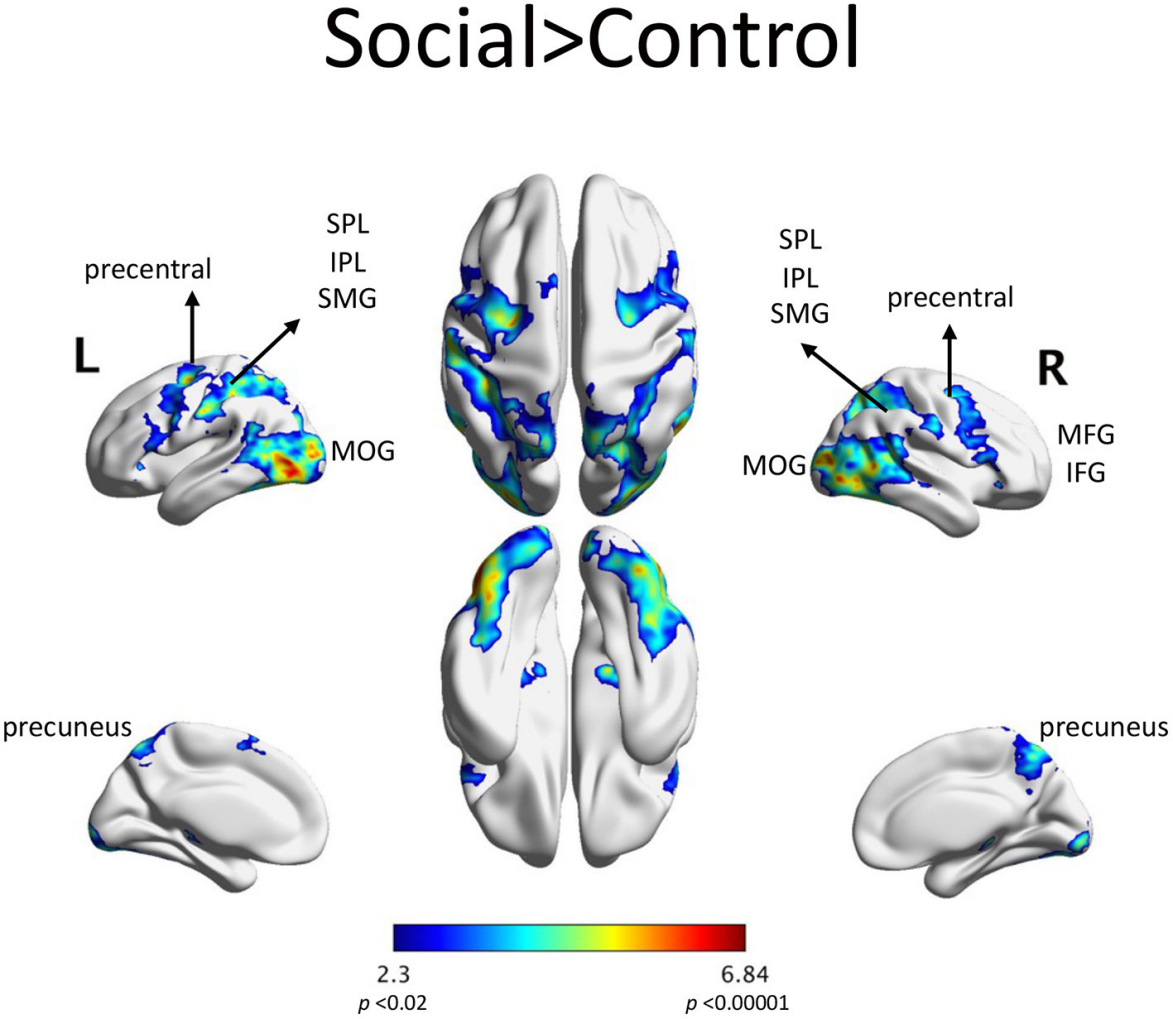
fMRI activations in the Social > Control contrast included: SPL: superior parietal lobule, IPL: inferior parietal lobule, SMG: supramarginal gyrus, MFG: middle frontal gyrus, IFG: inferior frontal gyrus, MOG: middle occipital gyrus. This activation was composed of largely one contiguous set of regions, with the highest activation in the left visual area. AAL regions with at least 1% of the cluster's volume are listed in Supplemental Table S1.

Scenarios depicting social interactions (Social condition) elicited fMRI activity in parietal regions: the bilateral superior and inferior parietal lobules, the bilateral supramarginal gyri and the precuneus. Increased activity in the Social condition was also seen in frontal regions: bilateral inferior frontal gyri, bilateral middle frontal gyri and bilateral orbital frontal gyri (Figure 3). The list of significant activations can be found in Supplementary Table S1.

#### MEG results

**Figure 4:**
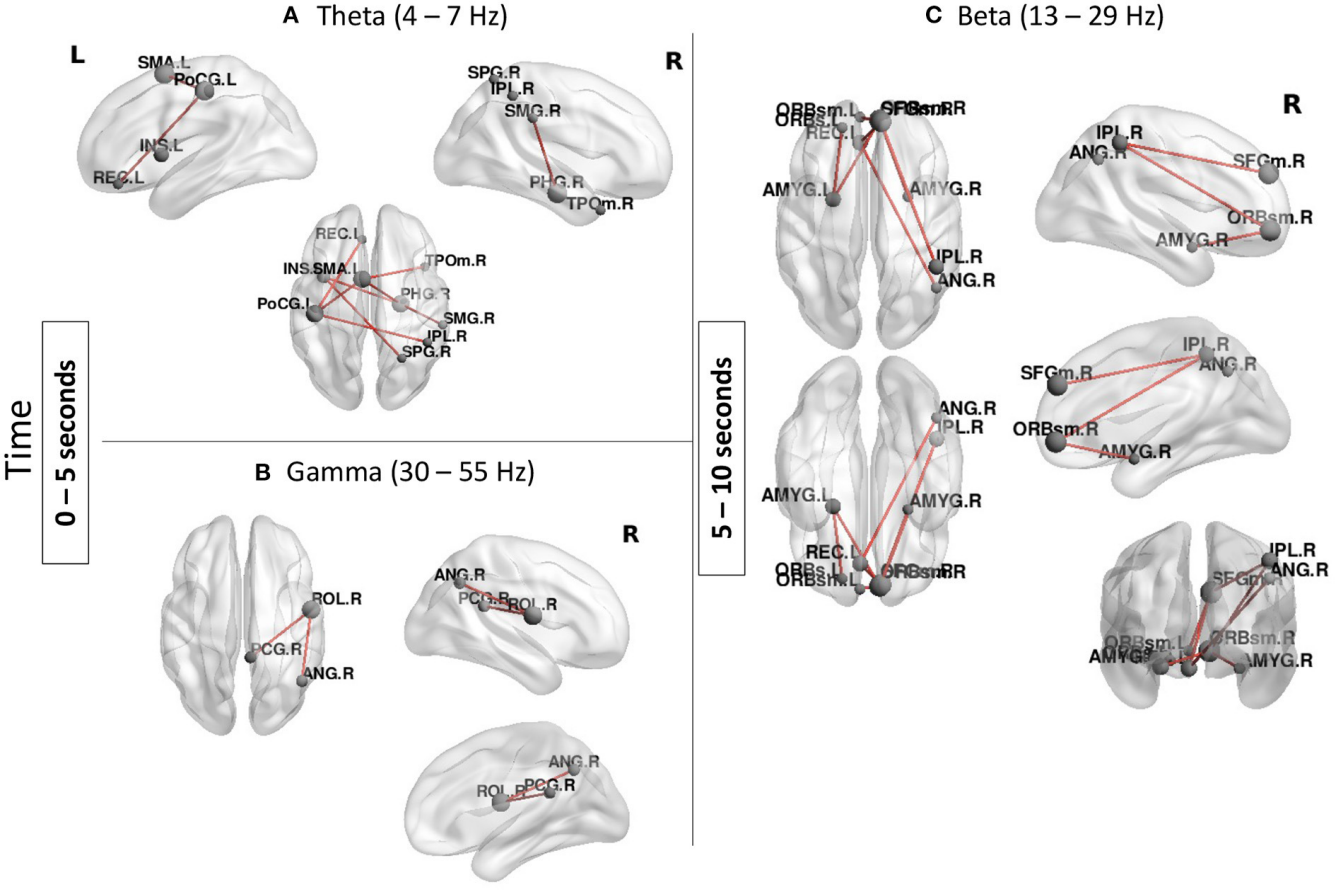
Functional connectivity results during the Social condition (Social > Baseline). **(A,B)** A temporal-parietal network was found in theta and gamma bands from 0 to 5 s. **(C)** A frontal-parietal network was found in beta band from 5 to 10 s. Node size represents the number of connexions each node has with other regions of the network, larger nodes indicate more connexions. Networks shown are reported at *p*_FWER_ < 0.05.

MEG results confirmed involvement of several regions found in the fMRI analysis but also suggest that parietal and frontal regions are involved in a sequential order rather than in concert in ToM processing (Figure 4). In the earlier time window: from 0 to 5 s, two temporal-parietal networks were involved, one in theta band (4–7 Hz) and one in gamma band (30–55 Hz). In theta band, the network was comprised of the right supramarginal gyrus, right superior parietal gyrus, right inferior parietal lobule as well as right temporal pole and left insula. Other regions which are not classically involved in ToM were also found in this network including the left post central gyrus and left supplementary motor area. In gamma band, the network included the right angular gyrus and posterior cingulate gyrus.

From 5 to 10 s, a beta band network involving mainly frontal parietal regions as well as subcortical structures was found in the Social condition. Regions in this network included the right angular gyrus and the right inferior parietal lobule and the bilateral amygdalae as well as the superior frontal gyrus and bilateral orbital frontal gyri. There were no significant networks found in alpha band or the last time window (10–15 s).

## Discussion

A large body of research suggests that humans have an intrinsic ability to attribute human experiences such as personality traits, relationships and thoughts to moving shapes based on their kinetic features (Heider and Simmel, 1944; Castelli et al., 2000; Schultz et al., 2003). Consistent with prior research, we found that adults made significantly more behavioural, perceptual, emotional and cognitive attributions in the Social compared to the Control condition. During the Social condition, we found recruitment with fMRI of the classic ToM network including the TPJ, superior temporal sulcus and precuneus, consistent with other studies using a similar experimental paradigm (Castelli et al., 2000; Osaka et al., 2012). These regions are also commonly involved in other ToM tasks such as those invoking false belief and social storey protocols (Carrington and Bailey, 2009). Our MEG results complemented the fMRI data by offering novel insights into the timing of the involvement of these regions, as well as the oscillatory frequencies that support these networks, adding exciting new information about the mechanisms of ToM processing. Specifically, our study extends the previous literature by suggesting that ToM is supported by processing first in a network comprised mainly of temporal-parietal regions followed by a network comprised of frontal-parietal regions. Based on the previously documented functions of regions that comprise these two networks, social attribution tasks involve an initial shifting of attention to the agent to which attribution is made (temporal-parietal connexions) followed by self/other delineation (frontal-parietal connexions). We also found that ToM processing is supported by neural oscillations in theta, beta and gamma. The current study highlights the complex interplay between neural activity, neural oscillations and specific timing of these activations to support ToM processing in healthy adults.

### ToM involves distinct temporal-parietal and frontal-parietal connectivity networks

Over the last few decades, several fMRI studies have tried to delineate the role of regions that comprise the ToM network. This network consists of the medial prefrontal cortex (*mPFC*), medial orbital frontal cortex (*mOFC*), the anterior cingulate cortex (*ACC*), the precuneus, bilateral temporal poles (TP), posterior superior temporal gyri (*STG*), bilateral temporal parietal junctions (*TPJ*), and bilateral inferior frontal gyri (*IFG*) (Molenberghs et al., 2016). Our MEG results help address this issue of priority of these brain areas. We found that networks from 0 to 5 s in the Social condition involved the right TPJ, right temporal pole, right parahippocampal gyrus and left insula. Similarly, a previous study using MEG, found that during false belief processing (ToM task), the right TPJ was recruited from 150 to 225 ms, followed by activation in the precuneus, the right inferior frontal gyrus (200–375 ms) and the superior frontal gyrus (300–400 ms) (Mossad et al., 2016). These findings corroborate previous suggestions that the right TPJ is one of the key regions involved in ToM processing to facilitate orienting to socially relevant stimuli. We also found that the TPJ was functionally connected with the insula, which would facilitate detection of salience of the socially relevant events in the videos. Parietal-temporal connexions were also found between the right TPJ and the right parahippocampal gyrus. Connexions between parietal to medial temporal structures support the hypothesis that previous knowledge stored in memory is accessed during ToM processing to help explain the relation between the mental state attribution and the action to be predicted (Frith and Frith, 2003).

In the second time window (5–10 s), a network with dense frontal-parietal connexions was found. The main frontal structures involved were the superior frontal gyrus and the right orbital frontal cortex, concordant with previous literature on the importance of medial frontal areas in ToM. The fact that this frontal region was seen at 5–10 s but not 0–5 s suggests that it follows the TPJ and the temporal-parietal networks. The fact however, that the TPJ and the prefrontal regions were functionally connected during ToM processing supports the integration of these key structures in the concerted processes of inferring the mental states of others and decoupling one's own mental state from others' mental states.

### ToM is supported by theta, gamma and beta oscillatory frequencies

The ToM networks we found were supported by theta and beta frequency bands in addition to a focal network in gamma band. Theta (4–7Hz) classically underlies long-range communication in the brain and facilitates complex cognitive processing (Mellem et al., 2013; Kaplan et al., 2017). The largest network we found was in the 0–5s time window in theta and indicated the early activation and integration of the temporal-parietal brain areas involved in ToM. Consistent with this, an EEG study using an animated videos paradigm showed that theta frequency oscillations were modulated by varying the complexity of the presented social interactions (Blume et al., 2015). In the same time window, there was a focal network in the right temporal-parietal region, in gamma. Gamma band activity is related to visual attention and awareness (Tallon-Baudry, 2009) and is central to the binding of perceptual representations with emotional meaning (Garcia-Garcia et al., 2010; Martini et al., 2012; Safar et al., 2020). This gamma band network thus suggests that the detailed attentional processing of the social, emotional aspects of the videos were being processed in this small, discrete network in the first few seconds of the video.

In the following 5 s (5–10 s), the third network was seen in beta band, anchored in the orbital, medial frontal areas. Frontal beta oscillations play a key role in top-down attentional control of information (Richter et al., 2018; Riddle et al., 2019; Kajal et al., 2020) and are associated as well with processing of visual, emotional stimuli (Güntekin and Başar, 2010; Luckhardt et al., 2017). This suggests that this beta network, underpinned the attentional processing of the social and emotional aspects of the videos. The fact that it involved the frontal regions, unlike the networks at 5–10 ms, strongly suggests that the medial prefrontal activation seen in fMRI *follows* the activity in the TPJ and other temporal parietal areas. Interestingly, however, this network included the right TPJ areas and amygdalae, thus linking the processing of the self vs. others, seen in these frontal areas, with the inferences of mental and emotional states, that may rely more on the TPJ and amygdalae. This also further highlights the hypothesis that ToM is not a singular process (Schaafsma et al., 2015) but rather is based on multiple cognitive subprocesses. We add to this by showing the sequence of these processes.

We had also predicted involvement of alpha band in the current paradigm as it was previously implicated in ToM processing (Perry et al., 2010, 2011) and was also found to be involved in the social attribution task in MEG in typically developing children (*n* = 43) (Mossad et al., 2017) but a reason why we may have failed to make this findings is the relatively small (*n* = 25) sample size in this study.

### fMRI source localisation vs. MEG functional connectivity analyses

Based on previous fMRI findings, it is known that there is an overlap across ToM study paradigms in core brain activations. In this study, the temporal-parietal junction, medial prefrontal cortex, inferior parietal lobule and the precuneus were found to be more highly activated in the social compared to the physical (control) condition in the source localisation fMRI analysis, which was previously reported in several ToM paradigms (see meta-analysis by Schurz et al., 2014). In the social animations paradigm, there is overlap with regions found in previous studies in the precuneus, medial prefrontal cortex, temporoparietal junction, superior temporal sulcus and occipital gyrus (Castelli et al., 2000; Moessnang et al., 2020; Schurz et al., 2021).

One of the unique aspects of this study design was the use of two neuroimaging modalities: fMRI and MEG. Since the focus of the analyses were different in each modality, a direct comparison is not supported; however, some conclusions can be drawn from each analysis. For example, we found regions that were recruited both in the spatial localisation analysis (done in fMRI) and the functional connectivity analysis (done in MEG). These regions included the right supramarginal gyrus (region in the right TPJ), the right superior parietal lobule and the right inferior parietal lobule. The consistent involvement of these regions in spatial localisation and functional connectivity highlights the previously hypothesised role of this right parietal region as being central to ToM. Damage to the right parietal cortex may therefore have the greatest impact on ToM processing, as our findings suggest that this region is not only recruited for ToM but is also communicating with other regions to process information about others. In a recent study investigating ToM skills in tumour patients, Campanella et al. (2022) found that patients with right superior parietal damage had the highest selective impairments in intention attribution compared to patients with temporal or frontal lesions both before and after surgery.

Importantly, the fMRI activations were largely bilaterally symmetrical, whereas the MEG hubs were right-dominant. Considerable research has suggested that the ToM, social-cognition network is right lateralised (Saxe and Kanwisher, 2003; Saxe and Wexler, 2005), consistent with the MEG findings. Since fMRI and MEG capture different processes (Hall et al., 2014), these differences in lateralization are unsurprising. Because fMRI relies on the slow hemodynamic response, it is biassed towards long-lasting processes (or neural activations) that occur in the very slow oscillatory frequency ranges, whereas, MEG will capture fast-occurring and high frequency activity. For this reason, differences in localizations including laterality can differ. The bilateral effects seen in fMRI suggest that with time, homologous brain areas are also activated, but the MEG results suggest that only the right lateralized parietal regions are functionally connected with other ToM regions during processing the social information in the videos.

It is also significant to note that some regions were found in the functional connectivity analysis in MEG but not in the spatial localisation analysis in fMRI such as the superior frontal gyrus, right temporal pole, left insula, bilateral amygdalae. This is not surprising as key structures, which may activate only for short periods of time, would be missed by fMRI. As some previous fMRI studies have shown involvement of these frontal and subcortical regions (Gallagher et al., 2000; Phelps and LeDoux, 2005; Gobbini et al., 2007), this suggests that their activation may also be task dependent. These data would suggest that the greater temporal resolution of MEG allows for greater sensitivity in identifying key hub regions implicated in ToM which are more transiently active, which would be not uniformly present in fMRI results.

## Future directions and limitations

A methodological advantage of this task is that does not require language proficiency and therefore the paradigm can be studied across developmental groups and clinical populations. For example, in a recent MEG study using this paradigm, we found a similar frontal-parietal network in beta band in healthy full-term born, 8-year-old children compared to preterm born children (Mossad et al., 2021), suggesting that this network may be recruited across typical development. We have also shown that this protocol can distinguish types of social interactions portrayed in the video in children with and without neurodevelopmental disorders (Vandewouw et al., 2021). However, a methodological limitation of this task is that given the length of each trial, fewer scenarios overall can be used in the social attribution task and therefore the results of this study are limited to the scenarios presented. Future studies can aim to compile a database of social attribution videos and investigate mental state attribution across various social interactions to allow presentation of a more standardised set of stimuli. Twenty-five participants completed the paradigm in both the MEG and the fMRI. A potential limitation is that there may have been practise effects in the fMRI due to the stimuli having been presented already in MEG. Activation in ToM regions were found in the fMRI but future studies should include measures such as eye tracking to ensure that the participants focus on the scenarios or use slightly different scenarios that are known to elicit similar descriptions. An important point for this study is that we have demonstrated that this protocol is adaptable to both neurophysiological and hemodynamic neuroimaging approaches. Clearly the MEG provides richer data and offers advanced options in understanding the timing and frequencies associated with various aspects of ToM processing.

## Conclusions

In this study, we found that ToM processing was supported by three networks in MEG in theta, beta and gamma bands. These networks included regions that are classically involved in ToM studies (Carrington and Bailey, 2009). The right TPJ was involved in all three networks, further highlighting its role as a key player in ToM processing, while medial prefrontal cortex and subcortical connexions were found only in beta band. The specificity of the findings was greater in MEG than fMRI; fMRI results showed bilateral, classic areas of activation only. Information about the temporal and oscillatory properties of these network dynamics through MEG allowed us to conceptualise a sequence for ToM processing, providing rich information against which atypically developing populations can be compared.

## Data availability statement

The raw data supporting the conclusions of this article will be made available by the authors, without undue reservation.

## Ethics statement

The studies involving human participants were reviewed and approved by SickKids Research Ethics Board, Hospital for Sick Children, Toronto, Canada. The participants provided their written informed consent to participate in this study.

## Author contributions

SM collected and analysed the data and wrote the manuscript. MV and KV helped analyze the data and contributed to manuscript development. EP and MT contributed to the design of the study and manuscript development. All authors contributed to the article and approved the submitted version.

## Funding

This work was funded by Canadian Institutes of Health Research (MOP-137115) to MT.

## Conflict of interest

The authors declare that the research was conducted in the absence of any commercial or financial relationships that could be construed as a potential conflict of interest.

## Publisher's note

All claims expressed in this article are solely those of the authors and do not necessarily represent those of their affiliated organizations, or those of the publisher, the editors and the reviewers. Any product that may be evaluated in this article, or claim that may be made by its manufacturer, is not guaranteed or endorsed by the publisher.
