# Supplementary material for: Characterising the spatial and oscillatory unfolding of Theory of Mind in adults using fMRI and MEG
Source: Front Hum Neurosci. 2022 Sep 20;16:921347. doi: 10.3389/fnhum.2022.921347 (PMC9530400; doi:10.3389/fnhum.2022.921347)
Supplement: Supplementary file 1 [file Table_1.DOCX]

Table S1 fMRI activations for the contrast: Social > Control

| Cluster number | # Voxels | p-value | Maximum Z-vlaue | X (mm) | Y (mm) | Z (mm) | AAL regions* |
| --- | --- | --- | --- | --- | --- | --- | --- |
| 1 | 35273 | 0 | 672 | -46 | -72 | 4 | MTG(Right), MOG(Left), PreCG(Left), MTG(Left), PoCG(Left), MOG(Right), IPL(Left), PoCG(Right), SPG(Left), PCUN(Right), PreCG(Right), IFGo(Right), FFG(Right), STG(Right), IFGt(Right), SPG(Right), PCUN(Left), IFGt(Left), IOG(Left), FFG(Left), SMG(Right), SOG(Right), ITG(Right), IFGo(Left), SMG(Left), SMA(Left), IOG(Right), ORBi(Left), IPL(Right), MFG(Right), SFGm(Left), MFG(Left) |
| 2 | 287 | 00131 | 543 | 16 | -30 | -2 | THA(Right), HIP(Right), LING |
| 3 | 274 | 00172 | 443 | -14 | -34 | -4 | THA(Left), HIP(Left), LING(Left), PHG(Left) |

*MTG= middle temporal gyrus, MOG= middle occipital gyrus, PreCG=Precentral Gyrus, PoCG= Postcentral gyrus, IPL=inferior parietal lobule, SPG=Superior parietal gyrus, PCUN=precuneus, IFGo= inferior frontal gyrus opercularis, FFG= fusiform gyrus, STG=superior temporal gyrus, IFGt=inferior frontal gyrus triangularis, IOG=inferior occipital gyrus, SMG=supramarginal gyrus, SOG=superior occipital gyrus, ITG=inferior temporal gyrus, SMA=supplementary motor area, ORBi=inferior orbital frontal gyrus, MFG=middle frontal gyrus, SFGm=medial superior frontal gyrus, THA=Thalamus, HIP=Hippocampus, LING=Lingual, PHG=parahippocampal gyrus
